# Urinary Semeiology, or Treatise on the Changes of the Urine in Disease, &c.

**Published:** 1843-10

**Authors:** 


### Art. III.

*Semeiotique des Urines, ou Traité des Altérations de l'Urine dans les Maladies; suivi d'un Traité de la Maladie de Bright aux divers ages de la vie.* Par Alfred Becquerel, m.d. &c.—*Paris*, 1841. 8vo, pp. 576.

*Urinary Semeiology, or Treatise on the Changes of the Urine in Disease, &c.* By Alfred Becquerel, m.d.—*Paris*, 1841.

The volume of which we have just transcribed the title does not now make its first appearance in this Journal. We some while since, in an earlier number, (26th of April, 1842,) introduced the second division of the work to the notice of our readers,—that devoted to the consideration of Bright's disease. Circumstances before alluded to prevented us at that time from analysing the first division; these circumstances no longer interfere with the discharge of an agreeable duty, that of making English observers of urinary disease acquainted with some of the most elaborate, and in a particular direction the most excellent researches, yet undertaken on the chemical constitution and pathological relations of the urine.

M. Becquerel's inquiries and results are collected under three heads. To the first of these is referred the study of the chemical and physical properties of the urine; here are examined the elements constituting it, and especially the variations in respect of quantity which it undergoes in disease. Next the physical properties of the fluid are reviewed, and the degree to which the chemical constitution can be inferred from the physical aspect carefully considered. In the second of these parts the scattered facts of the first are brought together, as elements for the establishment of the general propositions relative to the pathology of the urine. In the third part the author endeavours to apply the general principles thus ascertained to special pathology, and follows the modifications of the urine in different diseases in particular. Of each of these parts we shall give as full an account as the nature and importance of the subject justifies.

*Chemical and physical properties of the urine.* The old division of urines voided at different periods of the day and under different circumstances of fasting, repletion with food, &c., which was established by the early observers and retained by their followers, is rightly regarded as practically just by M. Becquerel. The *urina potûs*, the urine of digestion, and the urina sanguinis, are fluids which, though essentially the same, yet vary in a sufficient number of particulars to make it necessary we should in every case be aware to which of those kinds any given specimen belongs. To chemists having neglected to acquaint their readers of this circumstance, some of the questionable and many of the contradictory statements afloat on the chemistry of the urine may be attributed. An instance of this is furnished, according to M. Becquerel, by the quantitative analysis of Berzelius, which has so often been quoted in all tongues and lands, as the dictum of the great authority; here the quantity of urea and uric acid is certainly greater than it would have been, had the eminent analyst taken the mean of the amounts furnished by the three kinds of fluid. But this state of things prevails only in health. In various forms of disease which are capable of effecting change in the composition of the urine, it may be affirmed generally that the distinction in question ceases to exist; the only very notable exception occurs when a very large quantity of fluid has been gorged during the twenty-four hours. But although the urine is in these maladies, except in really exceptional instances, similar in properties at whatever time it be voided, the author generally selected the morning urine (urina sanguinis) for his experiments, because at that period its composition bears a closer relation than at any other to the constitution of the blood, and because then, if collected under the same circumstances, it is always identical. Such are the views which have led all physicians to choose the morning urine for the subject of experiment; but as M. Becquerel very fully shows, and as we are ourselves quite aware, this mode of proceeding is incapable of furnishing answers to all the questions that the practical physician may put himself. For example, the urine of a subject labouring under intense febrile reaction is denser, more acid, of deeper colour than that of a healthy person, and often presents a uric acid sediment combined with colouring matter; such urine appears to contain more uric acid therefore and animal matter than the healthy secretion: but this augmentation may be apparent and not real. Who can affirm that the case is not one of simple diminution of the proportion of water, and consequent concentration of the solid ingredients? M. Becquerel well argues that the affirmation cannot be made from a consideration of the morning urine only.

Another method of avoiding the errors which are liable to arise from examining the urine voided at a particular period only of the day was pointed out by Chossat, and employed by Lecanu in his very excellent experiments. We mean that of collecting all the urine passed in twenty-four hours, mixing it together and analysing the whole; but even this plan, adopted by the author in many instances is, he points out, liable to entail some kinds of error, and attended with much difficulty. He in the first place observes that it cannot be considered in accordance with sound principles of physiology to divide time into periods of twenty-four hours in reference to secreted products, that a subject may pass more urine one day, less the following, &c., and thus a state of compensation be effected. Lecanu, cognizant of this, examined the urine of six, eight, ten, and some twelve days, and then took the mean of the total results: but this mode of proceeding, though excessively laborious, is at least possible in the examination of the excretion of healthy persons, but it is impossible and open to important fallacy in the state of disease, as the urine alters in character from day to day. A difficulty encountered on the part of patients is, that it is very difficult to persuade them to keep all their urine, more especially to make sure of their always passing the contents of the bladder before those of the rectum, when about to have a stool. A further difficulty arises from the absolute impossibility of collecting the urine of subjects passing it involuntarily, and of others placed in various circumstances, which will readily suggest themselves.

M. Becquerel commences his chapter on the composition of the urine considered generally, by making the following statement: "The urine voided in the course of twenty-four hours, or that passed in the morning, by the same individual, is never of the same composition identically on any two consecutive days or occasions; the mean quantity of each chemical element is never precisely the same." Generally speaking, the variations in the quantity of each element are trifling in amount, but they may become seriously large. For example, M. Lecanu found that the quantity of urea passed by the male in twenty-four hours might vary between 22·446 grains and 9·119 grains, and by the female between 17·983 and 9·881 grains; in the analytical table which we shall presently transcribe, the author gives the mean of the oscillations which he, as Lecanu, observed in the proportions of the different ingredients. And there is an important inference from this, when the quantity of any principle is found in specimens of urine voided by persons out of health, to be but slightly above or below the mean standard, namely that the deviation discovered must be considered one compatible with a healthy constitution of the fluid.

TABLE,

Exhibiting the Mean Composition of the Urine of the Male and Female in the normal state, and the Mean deduced from the addition of both. The Table is constructed from the results of the Analyses of 8 healthy specimens of Urine: 4 of Males and 4 of Females.

| Chemical Elements. | MALES. | | FEMALES. | | GENERAL MEAN. | |
|---|---|---|---|---|---|---|
| | Urine of 24 hours. | Composition in 1000 parts | Urine of 24 hours. | Composition in 1000 parts | Urine of 24 hours. | Composition in 1000 parts |
| Quantity of urine | 1267·3 | | 1371·7 | | 1319·8 | |
| Density | 1018·900 | | 1015·120 | | 1017·010 | |
| Water | 1227·779 | 968·815 | 1337·489 | 975·052 | 1282·634 | 971·935 |
| Other matters beside water furnished by direct evaporation | 39·521 | 31·185 | 34·211 | 24·948 | 36·866 | 28·066 |
| Urea | 17·537 | 13·838 | 15·582 | 10·366 | 16·555 | 12·102 |
| Uric acid | 0·495 | 0·391 | 0·557 | 0·406 | 0·526 | 0·398 |
| Fixed salts not decomposable at a red heat. { Chlorides, Phosphates, Sulphates, or { Lime, Soda, Potass, Magnesia, | 9·751 | 7·695 | 8·426 | 6·143 | *9·089 | *6·919 |
| Organic matters, which cannot be isolated and calculated separately, { Lactic acid. Lactate of Ammonia, Colouring matters, Extractive matters, Hydrochlorate of Ammonia. | 11·738 | 9·261 | 9·655 | 8·033 | 10·696 | 8·647 |

* *Composition of the fixed salts in the Urine of* 24 *hours and in* 1000 *parts.*

| | Urine of 24 hours. | In 1000 parts. |
|---|---|---|
| Chlorine | 0·659 | 0·502 |
| Sulphuric acid | 1·123 | 0·855 |
| Phosphoric acid | 0·417 | 0·317 |
| Potassa | 1·708 | 1·300 |
| Alkaline and Earthy bases { Soda, Lime, Magnesia | 5·181 | 3·944 |
| | 9·089 | 6·919 |

The mean quantity of water voided in the urine of twenty-four hours being 1282·634 grains, M. Becquerel has found that it may vary between 800 and 1500 grains without exceeding or falling below "physiological limits." The inference from this is obvious and important: as the quantity of solid matter held in solution is the same whether the water be to the amount of 1200 or only 800 grains, the appearance of the urine must be very different in the two cases. And it might further be inferred from this, as experience proves is the fact, that in various species of disease the quantity of water does not swerve from the wide standard compatible with a healthy state of the fluid.

The quantity of water voided with the urine increases beyond the natural amount when the quantity of liquids drunk is considerable; thus two litres (about two quarts and half a pint) swallowed by a person habitually passing 1000 grains increased this average quantity to 2712·924 grains. In polydipsia (diabetes insipidus), in diabetes, and in hysteria and other nervous conditions, the quantity of water passed is greatly increased, as is well known.

Diminution of the quantity of water is of more common occurrence, and is referrible, according to the author, to the following causes. 1. The existence of fever, and consequently of all circumstances capable of giving rise to febrile reaction, such as acute and chronic inflammations. 2. Diseases of the liver and heart, especially if these are of a kind to produce general disturbance of the system. 3. Diseases, of whatever kind they may be, attended with such general disturbance. 4. The existence of abundant sweats. 5. The quantity diminishes immediately before death; complete suppression even may ensue at this period.

The proportional quantity of water in 1000 parts of urine oscillates slightly above and below 971·934 grains; it increases in chlorosis and anemic affections generally: in the former malady it may reach 990·298 grains. On the other hand, the author has sometimes found the proportional amount fall, in the febrile state, or during the paroxysms of pulmonary emphysema or cardiac disease, to 955 grains; the lowest amount met with, was 948· grains, observed in a strong woman labouring under milk-fever.

The sum of solid principles held in solution in the state of health in the urine is as follows:

| | Water. | Solid principles secreted in 24 hours. |
|---|---|---|
| Males ... | ... 1227·779 | ... 39·521 |
| Females ... | ... 1337·489 | ... 34·211 |
| General mean | ... 1282·634 | ... 36·866 |

The quantity may vary in the male between 36 and 41 grains, without ceasing to be compatible with health; in the female between 32 and 36. The conditions determining an unnatural increase are: 1. A rich, abundant, and strongly nitrogenized diet. 2. The consumption of an excessive quantity of water;—to this very important point we shall by and by return. 3. The existence of the disease polydipsia, almost as a consequence of the last. 4. Occasionally the urine voided under the influence of nervous affections, hysteria, &c., are characterized by containing sufficient solid ingredients to cause the total amount of these in the twenty-four hours to be unnaturally great. 5. Diabetes.

On the other hand, the circumstances productive of disease are: 1. The existence of febrile action, of acute inflammation, of functional disturbances, if to a marked amount, of attacks of disease of the heart, of the lungs and liver, &c. 2. If in addition to these states there be some cause of debility, of exhaustion, or of anemia present, the degree of diminution is much greater. 3. In chlorosis, anemia, and various states of debility, the same effect in the solid materials is sometimes observed; the contrary is the case in other instances, as we have just seen. The total amount of solids held in solution sometimes remains natural in disease.

In the natural state the quantity of urea varies as follows, according to the density of the urine and the sex of the subject:

| | Density. | Quantity of Urea in 1000 parts. |
|---|---|---|
| In Males ... | ... 1018·900 | ... 13·838 |
| In Females ... | ... 1015·120 | ... 10·366 |
| General mean | ... 1017·010 | ... 12·102 |

The quantity of urea voided in twenty-four hours may vary between 15

in females and 18 in males; it may very rarely exceed this amount in the state of disease; M. Becquerel himself has never observed such augmentation. Diminution of its sum is the general law in the morbid state, and in some cases may be so great as to lower the quantity voided in twenty-four hours to less than a third of the lowest amount compatible with health. A very valuable table is here given, displaying the condition of the urine in respect of density, and its amount of urea in 37 cases of disease arranged in five different series. The first series (we can only give the general results,) composed of cases of which the urine was in general dense, high-coloured, very acid, and moderate in quantity, (febrile cases, &c.); the mean density was 1021·914; the mean quantity of water 616·495 grains, of urea 9 grains. The second series comprises urines generally of lightish colour, of low density, and moderate abundance, furnished by young chlorotic subjects, by anemic patients, &c.; the mean density being here 1011·837; and the quantity of water 1161·390, the urea amounted to 7 grains only. The third series, composed of patients worn out by disease, by discharges of some kind, or by treatment (in one case of chronic pleurisy 200 leeches had been applied within a short time), and also labouring under febrile action, gives a mean density of 8014·880, as the mean quantity of water 594·051, and of urea 4·918 grains. In the fourth series are placed the cases of subjects resembling those of the first in every respect, except that the quantity of water voided by them was not only not diminished, but sometimes increased. The mean density in this series was 1010·500; the mean quantity of water 1285·230 grains, and of urea 8·919. In the fifth series the author has grouped cases of disease in which there was neither fever nor very marked functional disturbance; they give a mean density of 1017·747; as the mean quantity of water 949·316 grains, of urea 12·351.

Among the observations made upon changes in the *quality* of the urea, we observe the following assertion, which is at very distinct variance with ideas generally received. If urine taken indiscriminately and heated to ebullition, become turbid, opaque, and give origin to a deposit of some abundance, this is solely composed of sub-carbonate of lime, and sometimes of magnesia, from which the elevated temperature has removed the excess of carbonic acid required to hold them in solution. The ordinary belief, taught too by all recent writers, is, that the deposit consists in these cases of phosphates. The proofs of his view given by the author are, that a drop of acid instantly dissolves the precipitate,—this does not prove, however, that the salts are not phosphates; secondly, that this solution is accompanied with disengagement of bubbles of carbonic acid.

In the chapter on uric acid, and the alkaline and earthy urates, the everlasting question of the composition of the amorphous sediments of acid urine is once more discussed. The author agrees with the notion promulgated by M. Quevenne, namely, that the sediments in question consist of uric acid rendered amorphous in consequence of its combination or mixture with animal matters—these matters he believes to be the colouring and extractive substances. "Collect these sediments," observes M. Becquerel, "wash them successively with cold distilled water and cold alcohol, and in this way you will remove the greater part of the associated foreign matters; examine the sediment under the microscope, and an amorphous powder alone presents itself; treat this matter with a small quantity of nitric acid, or, better, of hydrochloric acid, which does not dissolve uric acid, nor the urates at the ordinary temperature; in the greater number of cases there will be no change, the matter will remain amorphous as before: now, if it were composed of acid, alkaline, or earthy urates, no doubt the nitric or hydrochloric acid would have separated the base, and allowed the uric acid to crystallize." This would be a very convincing argument if a correct statement, but we find the author admits that sometimes the crystallization does take place, and we believe that we have frequently detected it ourselves. This, however, only proves that in such cases lithates are present; and the question is, in reality, one of frequency: or it may be put in this way, if in a single instance an amorphous sediment were shown to be non-convertible into uric-acid lozenges by hydrochloric acid, this would prove at all events that its component materials were not ordinary lithates.

The analysis we have now given of some portion—an extremely small one, however—of the author's observations on the chemical and physical properties of the urine, will suffice to show our readers the manner in which those observations were made, and in which their results are recorded. We consider it advisable to leave this division of the subject for the present, and devote such space as we have at our command to the more strictly clinical part of the work having reference to the various descriptions of change occurring in the urine in the state of disease.

The different kinds of urine met with in disease may be divided according to the writer into four grand classes, each of them presenting several varieties: these classes are the febrile, the anemic, the alkaline, the nearly natural.

(1). *Febrile urine.* The author attaches, he says, little importance to the *term* febrile, inasmuch as the condition of urine to which it applies is observed in diseases of the liver, of the heart, and under other circumstances quite unconnected with the presence of fever. The urine in question, nevertheless, most commonly, it would appear, coexists with a febrile condition. M. Becquerel divides febrile urines into three sub-classes: (*a*), Febrile urine properly so called, presenting itself in persons labouring under febrile action or intense functional disturbance, no matter what be the malady causing either: (*b*), Febrile urine occurring under conditions of debility; or in the same circumstances as the last variety, some cause of weakness, debility or exhaustion being superadded: (*c*), Febrile urine, in which the quantity of water in twenty-four hours has not been sensibly influenced, the individuals affected being in the same conditions as those of the first and second varieties.

(*a*). The mean of eleven analyses of febrile urine properly so called was as follows:

| | |
|---|---|
| Quantity of urine ... ... | 685·050 |
| Water ... ... ... | 660·364 |
| Matters in solution ... ... | 24·686 |
| Density ... ... ... | 1021·840 |
| Urea ... ... ... | 8·996 |
| Uric acid ... ... ... | 999 |
| Inorganic salts ... .. | 4·849 |
| Organic matters ... ... | 9·824 |

The concentration of the fluid signified by this general result is attended with increased intensity of colour and augmented consistence. Mucus and lithic acid impair its transparence; it sometimes contains a small quantity of albumen, but if so, temporarily. Excess of lithic acid does not always lead to precipitation spontaneously; cold or nitric acid are required to produce this effect. During the continuation of the same febrile action, and without any relation to the period of the affection, the urine may be one day transparent, the next turbid; the varying quantity of water accounts for these changes.

The essential condition for the production of this species of urine is febrile action, no matter what be the cause of this. Its intensity and duration united regulate the amount of the abnormal state of the fluid. And duration appears to be more powerful in its influence than intensity,—at least M. Becquerel states that this is the reason that it is not by any means rare (although the contrary may have been asserted) to find the urine natural during the paroxysm of intermittent fever, whatever be the stage during which it is voided. The same is true of continued fever, if it be of short duration and slight in amount.

The manner in which febrile action induces the diminution of water, diminution of solids, and increases uric acid characteristic of that state, is next investigated by the author. He hesitates not to ascribe the second-named change to the abstinence of fever-patients, which bears principally on the urea and inorganic salts non-decomposable by heat. The two other modifications appear to him due to congestion of the kidneys, a state of those organs of which he has repeatedly ascertained the presence in cases of various acute maladies, or chronic diseases attended with fever. Admitting this, the intimate nature of the influence exercised by congestion of the kidney in effecting the modifications referred to, is still a point to be determined.

(2). *Anemic urine.* We pass over the other varieties of the febrile class, and reach the chapter on the anemic species, of which the author admits two varieties—the anemic properly so called, and the concentrated anemic.

In anemic urine properly so called, the water is in about the natural proportion, or slightly diminished, the sum of matters in solution reduced considerably below the healthy standard; the urea, uric acid, and inorganic salts, likewise diminished, and the organic matter, properly so called, lowered in quantity also, but to a less extent. In point of quantity anemic urine is usually about natural; its density is lower than in the healthy fluid; its colour light, with a greenish hue; it never contains uric acid as a spontaneous sediment, or as one determinable by cold or nitric acid.

Among the numerous circumstances in which anemic urine is observed is the convalescence of acute diseases, especially where the patients have undergone considerable impoverishment of blood during the previous malady. Paleness, low density, and imperfect acidity, constitute natural conditions during convalescence, and such state is of better augury than if they are high coloured, dense, turbid, or strongly acid. These latter qualities denote the occurrence of fever in the evening or night, or the existence of some persistent lesion in the solids. In some cases, in the midst of a state of convalescence with its natural anemic urine, the sudden appearance of colouring matter with turbidity may be the first circumstance announcing a coming relapse, or the supervention of a near disease. A very interesting example of this kind is related, where variola supervened in an anemic young woman recovering from articular rheumatism.

(3). *Alkaline urine.* Alkaline reaction of the urine always results from decomposition of the urea. This decomposition may occur at the moment of secretion; as we have had occasion to mention in former Volumes, M. Rayer considers this to be the case in chronic nephritis, an opinion which the author regards as difficult of demonstration: certain it is that the urea does not usually undergo change until it has reached the bladder, where the presence of pus facilitates in some cases the chemical action necessary. The causes capable of determining alkalescence of the urine are: acute and chronic nephritis; Bright's disease in some cases; prolonged stagnation of the urine in the bladder; diseases of the bladder accompanied with secretion of pus; certain diseases of the brain and medulla spinalis. In a certain number of cases the cause of alkalescence cannot be traced.

The next chapter introduces us to the modifications induced in the urine by the existence of various general morbid states of the system. Great losses of fluid are among the most important of these states,—take the example of dropsies. It has generally been said that the urine in dropsy contains proportionably to its water (which is diminished) a greater amount than natural of solids. This notion requires qualification however; all dropsies are not identical in their effects on the urine. First, when dropsy (anasarca) occurs suddenly in individuals who have not undergone previous deterioration of strength, the urine does assume the above characters, those of febrile urine,—its colour becomes deep, it is turbid, sedimentary, and becomes loaded with lithic acid. Secondly, if the dropsy have been slowly established, and connected with any of the various states which determine impoverishment of the blood, the urine will possess the qualities of the anemic class. Thirdly, in dropsy connected with disease of the liver, the urine is febrile to a very high degree. Fourthly, in cardiac dropsy the urine varies in character extremely,—it may be pale or high coloured, transparent or turbid, sedimentary or without deposit,—albuminous or not. Lastly, in the dropsy attending Bright's disease, the general tendency of the urine is to assume the anemic type.

The points we have already explained lead to the inference that various conditions and circumstances must, during the course of each particular disease, tend to modify the qualities of the urine: scarcely exists there an affection which will not at different periods of its course, and under varying conditions of the patient's general state, be attended with a urinary discharge of different types. Hence the importance, nay, necessity, of examining the excretion at various periods of maladies.

M. Becquerel has not observed "that the urine remains clear and transparent in cases of disease terminating fatally, or that it becomes turbid and sedimentary in the event of a fortunate termination." He disagrees altogether with M. Martin Solon in his announcements respecting the "critical" character of albuminous and lithic acid sedimentary urine in acute maladies. M. Martin Solon in truth only observed, as the author, that certain sedimentary conditions of the urine occur during the course of acute diseases; and that he should have met such conditions more frequently in instances of recovery than of death, is a simple consequence of the fact that under ordinary circumstances acute diseases end more frequently by recovery than death. These are precisely the views which we four years ago (Brit. and For. Med. Rev., vol. VIII., p. 143, July, 1839,) took of the bearing of the statements made by M. Solon; and we cannot forbear from noticing how completely the experience of M. Becquerel, as referred to in several places of the present article, supports the ideas frequently advocated in this Journal respecting the utter groundlessness of the fashionable creed of "critical" discharges. The more matured our experience grows, the more firmly persuaded do we become that a greater delusion than this doctrine of "critical discharges" never sprang from *à priori* reasoning, careless observation, and love of the marvellous.

It is to be remembered too, that in estimating the qualities of the urine in certain diseases and in regarding them as dependent upon these, a broad source of error lies in the non-appreciation of the changes effected by the nature of the food, drink, and medicines ingested during sickness. It is only observers who, like him whose work we notice, bear in mind all such modifying circumstances, and who labour upon so vast a number of cases, that slight errors correct themselves in the general result,—it is only men of this stamp that deserve a patient hearing when they announce the issue of their toils. And when we see young gentlemen or old ones boiling a few drops of urine, or mayhap dropping nitric acid into the same a dozen or so times, and on the strength of experience in this wise besieging the editors of Journals with their "Chemico-practical" papers, or even manufacturing volumes thereupon, we cannot avoid a pitying smile at the vanity of these petty dabblers, nor at the same time help lamenting a state of literature which fosters the production of such outrageous abortions.

We have reached the Third Part of M. Becquerel's volume. Here are considered the modifications arising in the urine in the course of particular diseases. We shall select two of these for the subject of some remarks founded on the details given by M. Becquerel. These details are so close, so minute, and so full, that in themselves they will not bear condensation without risk being incurred of misinterpreting their laborious author's meaning; and our space is so limited that complete extraction of the history of any one disease is beyond our means.

*Scarlatina.* In connexion with the subject of scarlatina the true nature of the anasarca occasionally attending the convalescence or advanced periods of the disease is most luminously discussed. From the writer's researches, conducted at the Children's Hospital in Paris, he finds himself justified in inferring that a certain number of these dropsical affections (the proportion will be presently given,) are the consequence of Bright's disease, and that others cannot be legitimately referred to that cause. Among these dropsical affections some originate and advance after the manner of acute diseases; others follow a chronic course. The latter generally occur in enfeebled and anemic subjects; they may terminate by recovery of health, but in a considerable number of cases the patients succumb under the influence of the dropsy and of increasing debility, the fatal issue being in some instances hastened by an intercurrent malady. In all these cases the urine presented the following characters: colour of slight depth, sometimes clear, greenish, or at others a little turbid; slight acidity; density below the natural standard; diminution of the solid constituents (anemic urine.) And in one case when the dropsy was unconnected with Bright's disease, the author found a notable quantity of blood in the urine. Albumen was far from being constantly discoverable, and when it did exist it was only in small proportion and at an advanced period of the disease. Consequently it appears that albuminuria in these dropsical subjects instead of preceding the dropsy, as in Bright's disease, occurred only as a consecutive and secondary phenomenon in the limited number of cases in which it existed at all. Nevertheless there is a great and close degree of similarity between the two kinds of urine, that of the dropsy we are now speaking of and of Bright's disease; both are anemic, but the constancy and large amount of the albumen contained in the urine distinguish the latter. The diagnosis must be excessively difficult in some cases; impossible even, where the examination of the urine has not been undertaken until a certain time after the supervention of the dropsy, the small quantity and capriciousness of appearance of the albumen in the non-renal anasarca seems then the only circumstance capable of aiding us in the distinction. Yet it is most important to be able to distinguish the two causes, for the prognosis is materially different in them.

Children dying with dropsy after scarlatina, and without Bright's disease, present the following conditions on post-mortem examination: The subcutaneous and sometimes the deep intermuscular cellular tissue is infiltrated with serosity. The peritoneum, and in some subjects the pleuræ and pericardium, contains serosity, generally straw-coloured and transparent. All the organs are remarkable for their pallor and anemia; some of them infiltrated with serosity,—for example, in many cases the submucous cellular tissue of the coats of the intestine. The lung is almost always distinctly and considerably œdematous; the liver soft, flaccid, pale, and on incision giving issue to serosity. The kidneys present peculiar appearances, which are referrible to œdema of their substance, occurring in two degrees. In the first degree the kidneys appear harder and more swollen than natural; the capsule may be completely separated from them; under this the tissue appears of a dull white colour, (without any trace of yellow hue,) interrupted here and there with vascular streaks. The entire of the cortical substance, even that prolonged between the tubuli, appears of the same colour, and presents neither granular texture nor vascularity. The tubular substance presents its natural form, and by its reddish hue stands out in strong relief from the surrounding pallid ground. Pressure forces out a certain quantity of serosity from the altered tissue; and when this is removed, the kidney loses its swollen and turgid aspect, and becomes soft and flaccid. This change may affect the entire or a part only of the cortical substance; in the latter case, it would appear, especially the neighbourhood of the hilus.

In the second and more advanced stage, the kidneys are decidedly larger and harder than natural; the capsule continues easily separable; the cortical substance is of a slightly yellowish white colour, or perfectly white and shining. No vascular appearance is visible; serosity in greater quantity is expressible as before; the tubuli look paler, but still are obviously distinguished by colour from the cortical encompassing them. The affection is always spread over the entire of the two kidneys; and it can only be regarded as a very advanced evidence of the general tendency to dropsy, like the infiltration of the liver, &c. The albumen in the urine in such cases appears to be furnished by the serosity pervading the renal tissue, which makes its way by a sort of infiltration or transudation into the excretory passages; be this as it will, the author's cases justify him in the inference that in the present kind of dropsy, the urine only contains albumen when the kidneys participate in the general serous infiltration of the body. In some cases of dropsy after scarlatina, where the urine contained no albumen, the author found the kidneys slightly paler than natural but otherwise unaltered.

From all that has been said it follows that during the period of desquamation of scarlatina, either of these kinds of dropsy may develop itself. 1. Acute dropsy with fever, without albumen in the urine. This is manifestly the rarest form; the author has not himself had occasion to observe it. 2. Dropsy produced by Bright's disease. 3. Dropsy such as we have just with some minuteness supplied the author's remarks upon. This species he conceives to be indubitably the most frequent.

We shall close the subject of scarlatina with a tabular statement of the history of 31 cases of the disease observed by the author at the Children's Hospital or La Charité.

Of the 31 patients 16 died, 15 recovered; an amount of mortality which the author endeavours to account for by the unusual severity of the disease in the entire number of cases. Of the 15 cases of recovery, 3 only suffered from anasarca: in one of these cases there was no trace of albuminuria; in the other two the urine was strongly albuminous and the symptoms of Bright's disease present—in these recovery was much slower than in the other case.

In the 16 fatal cases:

6 were complicated with dropsy; as follows:

| | |
|---|---|
| 6 | 1 with no disease of the kidneys. |
| | 3 with œdema of the kidneys. |
| | 2 with Bright's disease to a marked amount. |

10 died without dropsy; as follows:

| | |
|---|---|
| 10 | 2 Bright's disease, acute, with albuminuria. |
| | 1 Suppuration of tonsils aud pneumonia. |
| | 1 Gangrene of tonsils. |
| | 1 Pseudo-membranous inflammation of the mouth. |
| | 1 Capillary bronchitis. |
| | 1 Bronchitis and inflammation of intestines. |
| | 1 Pneumonia. |
| | 1 Gastro-enteritis. |
| | 1 Cerebral disease. |

All these facts sufficiently prove the important point that congestion of the kidneys is far from being a constant condition in scarlatina. They likewise show that Bright's disease is a rare complication of the cutaneous affection (4 times in 31 cases.)

*Diseases of the spinal marrow.* We shall next condense the author's observations upon the condition of the urine in diseases of the spinal marrow. These diseases may, in respect of their relation to the urine, be divided into two classes: in the first of these there is no disorder of function either in the kidneys or bladder; in the second the urinary functions are more or less disturbed. 1. In the first class of cases the urine presents no deviations from the natural condition; though there is always a tendency, from the persistence of the spinal disease, to the passage of the case into the second class. This is a very important preliminary point to determine; it shows that when modifications of the urine do attend this class of diseases, they are not the *direct* produce of the medullary affection, but immediately caused by some intervening condition.

2. In the second class of cases there is either (*a*) retention of urine, which is a rare phenomenon, and often accidental, or (*b*) involuntary discharge of the urine, which is on the contrary very frequent. Under such circumstances the urine is always altered in character; it becomes alkaline, of a dirty yellow colour, pale, and of about normal density. In other cases it becomes clearer and less dense than in health. Such urine almost always containsin suspension or solution all or some of thefollowing principles: abundant opaque mucus, muco-pus, or pus; a little albumen, especially in the latter cases; triple phosphate of ammonia and magnesia; pulverulent phosphate and carbonate of lime. Now is such urine a product of vitiated secretion of the kidneys, or is it due to the chronic inflammation of the bladder, which so often accompanies advanced disease of the spinal marrow? The author considers the question one not easy of solution; for when we consider that the paralysed condition of the bladder renders the escape of the urine from that viscus immediate, we should be tempted, à priori, to regard the urine as a direct product of renal secretion. Without doubt such is, too, the fact in some cases; and the kidneys have frequently been found diseased, to confirm the supposition. But the author is of opinion that more commonly the following are the real phenomena of the case. In the first place the evacuation of the contents of the bladder does not occur immediately upon these contents making their way into it; the bladder must first be filled and distended with fluid, and then any excess escapes. The urine stagnates in the blood consequently, and from this stagnation arises a spontaneous alteration of the fluid. Some authors deny the reality of such *spontaneous* change; M. Becquerel, admitting their correctness for a moment, at least contends that the repeated and continued stagnation of the urine must induce inflammation of the mucous membrane of the bladder. Once this cystitis is developed, the muco-pus and pus secreted, alter the urine and give it new properties. These notions are not new, as the reader will observe; but we are glad to find them adopted by so practical a writer as M. Becquerel: for we confess that to have the mere chemist talking of the purulent secretion acting as a ferment on the urine gives us but little satisfaction and confidence in the correctness of their doctrines. If the kidneys themselves become inflamed in consequence of extension of the disease from the bladder, they may, M. Becquerel admits, *secrete* alkaline urine.

We must now for the present (and for the second time) take leave of M. Becquerel's labours. It is but a small part of the encomiums to which these labours have justly entitled him to say, that his book is the best existing guide to the pathologist desirous of making himself familiar with the changes of the important fluid of which it treats, and anxious to learn the inferences in respect of diagnosis and prognosis derivable from a profound consideration of those changes. The work is conceived in a spirit of philosophy which redounds as highly to the honour of the writer's intellect, as the extraordinary labour, with which his researches have been conducted, clearly evince his deep love of truth and powers of industry.

